# Insights into two-phase flow dynamics in closed-loop pulsating heat pipes utilizing Fe_3_O_4_/water: experimental visualization study

**DOI:** 10.1038/s41598-024-67637-y

**Published:** 2024-07-17

**Authors:** Hamid Reza Goshayeshi, Seyed Borhan Mousavi, Saeed Zeinali Heris, Issa Chaer

**Affiliations:** 1 School of Safety Science and Engineering, Xi’an University of Science and Technology, 58, Yanta Mid. Rd., Xi’an, 710054 Shaanxi China; 2 Department of Mechanical Engineering, Mashhad Branch, Islamic Azad University, Mashhad, Iran; 3 J. Mike Walker ‘66 Mechanical Engineering Department, Texas A&M University, College Station, TX 77843 USA; 4 Faculty of Chemical and Petroleum Engineering, University of Tabriz, Tabriz, Iran; 5 School of Built Environment and Architecture, London South Bank University, London, SE1 0AA UK

**Keywords:** Pulsating heat pipes (PHP), Two-phase flow patterns, Flow oscillating, Liquid slug, Vapor plug, Engineering, Chemical engineering, Mechanical engineering

## Abstract

This article discusses a focused study on visualizing the flow patterns in a two-phase pulsating heat pipe (PHP) using Fe_3_O_4_/water as the working fluid at 3 V/V% concentration. The research also aims to meticulously examine phase change phenomena in the heating section, particularly focusing on bubble formation and expansion processes. A high-speed video camera was utilized to capture dynamic insights into the behavior of the Fe_3_O_4_/water mixture. Based on the findings, a straightforward model was developed to explain bubble generation and growth in the mixture, serving as a useful reference for future PHP designs and optimizations. Visual observations also noted the stable nature of the Fe_3_O_4_/water nanofluid over a 4-day period, confirming its consistency throughout the experiments. Moreover, the impact of heat load variation on the evaporator section was assessed using controlled heat inputs ranging from 10 to 80 W. Observations on the arrangement of slugs and plugs at a 50% filling ratio revealed interesting self-adjusting flow patterns in response to increasing heat inputs, providing valuable insights into PHP operational dynamics. Notably, the oscillatory flow behavior of Fe_3_O_4_/water, the chosen working fluid, exhibited greater activity in comparison to water. This distinctive flow behavior contributed to achieving heightened thermal performance efficiency for the Fe_3_O_4_/water system, attributed to its faster attainment of the annular flow condition.

## Introduction

Pulsating heat pipes (PHPs), also known as oscillating heat pipes (OHPs), are an innovative and emerging technology that has garnered significant attention since their proposal by Akachi^1^. This cutting-edge field of research focuses on exploring the potential of PHP/OHPs for various applications. PHPs are capillary-driven heat transfer devices with a closed-loop structure and a working fluid, like water or alcohol. They operate on two-phase heat transfer, using evaporation and condensation to transfer heat. PHPs are ideal for handling transient heat loads in applications such as electronics cooling and space systems, where heat dissipation needs fluctuate^2^. Performance of PHPs can be enhanced by optimizing the wick structure^3^, proper working fluid^4^, geometry and dimension^5^, start-up assistance^6^, coatings^7^, operating parameters^7^, etc. Over the past few years, researchers have given considerable importance to the choice of working fluids and how they impact the thermal efficiency of PHPs. The choice of working fluid is considered a crucial factor in determining the PHP’s efficiency in transferring heat. Scientists have explored various thermophysical properties of working fluids and proposed correlations to understand better and predict the heat transfer capabilities of PHPs^4^. The investigation conducted by Qu et al.^8^ evaluated the applicability of water and ethanol as potential working fluids within PHPs. The researchers concluded that the selection of a suitable working fluid should be customized to match the dimensions of each individual PHP. Xu et al.^9^ conducted a series of experiments using various ratios of water: ethanol as a hybrid fluid and nanofluids to examine how they impact the heat transfer characteristics of a PHP. Hybrid working fluids in PHPs, particularly those using FLG (Few-Layer Graphene) nanofluids, demonstrated superior thermal performance with an average enhancement ratio of around 25.16%. Furthermore, their investigation unveiled that the 30 V/V % ethanol–water blend exhibited the highest heat transfer performance compared to DI water, pure ethanol, and other ethanol–water compositions (20, 40, 60, and 75 V/V%)^9^.

The incorporation of nano additives has captured the interest of researchers aiming to enhance the heat transfer capabilities^10–16^ of working fluids in PHPs. This interest stems from the remarkable attributes of nano additives, including outstanding thermal conductivity and enhancement of convective heat transfer. Additionally, these additives prevent dry-out, offer a substantial surface area^17–20^, ensure stability^21–23^, and enable customization of heat transfer characteristics^24–26^. Various nanosized materials have been incorporated into base fluids to create high-heat transfer property nanofluids. Among these, metal oxide nanoparticles like alumina, iron oxide, and copper oxide are commonly employed in nanofluid preparation because they maintain stable dispersion in the base fluid^27–31^. Iron oxide (Fe_3_O_4_) nanoparticles are applied as nanofluids in PHPs due to their advantages, including high thermal conductivity, excellent stability, magnetic properties, and the ability to reduce thermal resistance^32–38^.

Riehl et al.^39^ employed a copper-based oscillating heat pipe (OHP) to effectively regulate the temperature of electronic components. The study compared the performance of the OHP using deionized water and a CuO-water nanofluid containing solid nanoparticles at a concentration of 3.5 wt.%. The OHP was filled to 80% of its internal capacity with the chosen working fluid and subjected to horizontal testing. Significantly, distinct and vigorous pulsations commenced at lower heat loads when utilizing the CuO-water nanofluid. This early slug/plug formation onset resulted in minimized temperature differentials between the evaporator and condenser right from the beginning. The introduction of the nanofluid played a crucial role by acting as nucleation sites, leading to enhanced vapor generation. This effect, in turn, led to heightened pulsation frequencies, even when operating at lower power levels. While the nanofluid improved OHP operation, it limited the device’s highest heat load due to additional pressure drops caused by increased viscosity; However, this limitation was not significant. An experiment was conducted by Alizade Jajarm et al.^34^ to evaluate the heat transfer improvement and effect of a magnetic field in three-dimensional pulsating copper heat pipes (3D-PHP) by introducing iron oxide nanoparticles dispersion in water. The proper filling ratio was identified as 50% to avoid “burnout.” According to their findings, using 2 wt.% of Fe_3_O_4_ nanofluid significantly enhanced the thermal performance of the PHP, particularly at a 50% filling ratio, surpassing pure water.

Moreover, a notable enhancement in the heat transfer coefficient by 15% was observed when employing a corrugated evaporator within a magnetic field, in comparison to the same corrugated evaporator operating without the magnetic field. Furthermore, utilizing nanofluid brought about significant improvements, with thermal resistance reductions of 18%, 20%, and 25%. Davari et al.^40^ explored the intricacies of a closed-loop oscillating heat pipe (CLOHP) employing Fe_3_O_4_ nanofluid/water as the chosen fluid. The investigation encompassed utilizing three distinct types of condensers to comprehensively explore their impact on heat transfer performance. They found that the nanofluid increased thermal performance, resulting in an average heat transfer improvement of approximately 11% when using Fe_3_O_4_/water nanofluid at a 2 wt.%. The horizontal corrugated condenser exhibited the lowest thermal resistance and highest efficiency compared to straight and vertical corrugated condensers, showcasing a novel achievement not previously reported. In a study by Jin et al.^41^, molecular dynamics simulations were employed to delve into the atomic and thermal characteristics of Fe_3_O_4_/water nanofluid within an OHP with copper walls. This investigation meticulously assessed the influence of varying percentages of Fe_3_O_4_ nanoparticles on crucial parameters such as density, velocity, temperature profiles, and heat flux over a simulated timeframe of 20 ns. The outcomes of the study revealed a distinct trend: with the increasing of Fe_3_O_4_ nanoparticle content from 1 to 4 wt.%, there was a concurrent elevation in the HF, transitioning from 1561 to 1602 W/m^2^. Coinciding with this, the maximum velocity displayed a decrease, shifting from 0.044 to 0.038 Å/ps. These findings shed light on the intricate relationship between nanoparticle concentrations and heat transfer characteristics, as elucidated by thorough molecular dynamics simulations. Table 1 summarizes the key findings of the conducted works.

**Table 1:** Summarized results of previously conducted works.

Working fluid	Used material	Fill ratio (%)	Conclusions	References
Acetone, methanol, ethanol	Copper	60–80	Acetone serves as a better working fluid in terms of high heat transfer rates at a 60% fill ratio	^42^
Water, ethanol	Copper	30, 40, 50, 70, 80	The CLPHP has a better thermal performance for water and ethanol at fill ratios of 40% and 50%	^43^
Acetone, methanol, deionized water	Copper	40–70	Methanol serves as a better working fluid at a fill ratio of 45% and heat input of 120 W respectively	^44^
FC-72 and R113	Silicon	41 and 58	The study demonstrated that evaporator, adiabatic, and condenser sections were largely occupied by annular slug-plug bubbly flows	^45^
Water, ferrofluid	Copper	N/A	Ferrofluid improves the efficiency of thermal performance; further, this study helps to improve electronic cooling devices more efficiently	^46^
Acetone, methanol, ethanol, heptane, distilled water	Copper	N/A	Heptane and Acetone exhibit better heat transfer characteristics among other working fluids	^47^

Conducted literature review highlights limited studies on visualizing PHP using nanofluids. Previous research has predominantly examined the heat transfer performance of PHP using copper or aluminum materials. Numerous researchers have conducted visualizations of the heating segment within a PHP, employing working fluids such as propane, HFC-134a, and butane^48–52^ as far as our understanding goes, there is a lack of research that specifically centers o flow visualization of Fe_3_O_4_/water. Furthermore, the opacity of materials like copper and aluminum presents a significant hurdle in observing the movement of the two-phase heat transfer fluid within the PHP, spanning from the evaporator to the condenser.

Consequently, the central aim of this research is to construct a glass prototype heat pipe (PHP) as a means to illuminate the inner workings of a PHP and to closely examine the behavior of two-phase flow within its evaporator, adiabatic, and condenser components across diverse heat loads. The comprehension of flow patterns intrinsic to PHPs holds pivotal importance, as it guides the customization of design for specific applications and the optimization of their heat transfer attributes.

In light of this, a dedicated visualization study focused on the flow pattern in a two-phase PHP fueled by Fe_3_O_4_/water as the designated working fluid. The investigation honed in on a meticulous assessment of phase change phenomena within the heating section, particularly accentuating the intricate processes of bubble inception and expansion. To accomplish this, the research employed a high-speed video camera, offering a dynamic glimpse into the dynamics of the Fe_3_O_4_/water mixture. Based on the results obtained from our visualization study, we developed a simple model to describe bubble generation and growth in the Fe_3_O_4_/water mixture. This model will serve as a valuable reference for future discussions and aid in designing and optimizing PHPs for various applications.

## Experimental section

**Figure 1:**
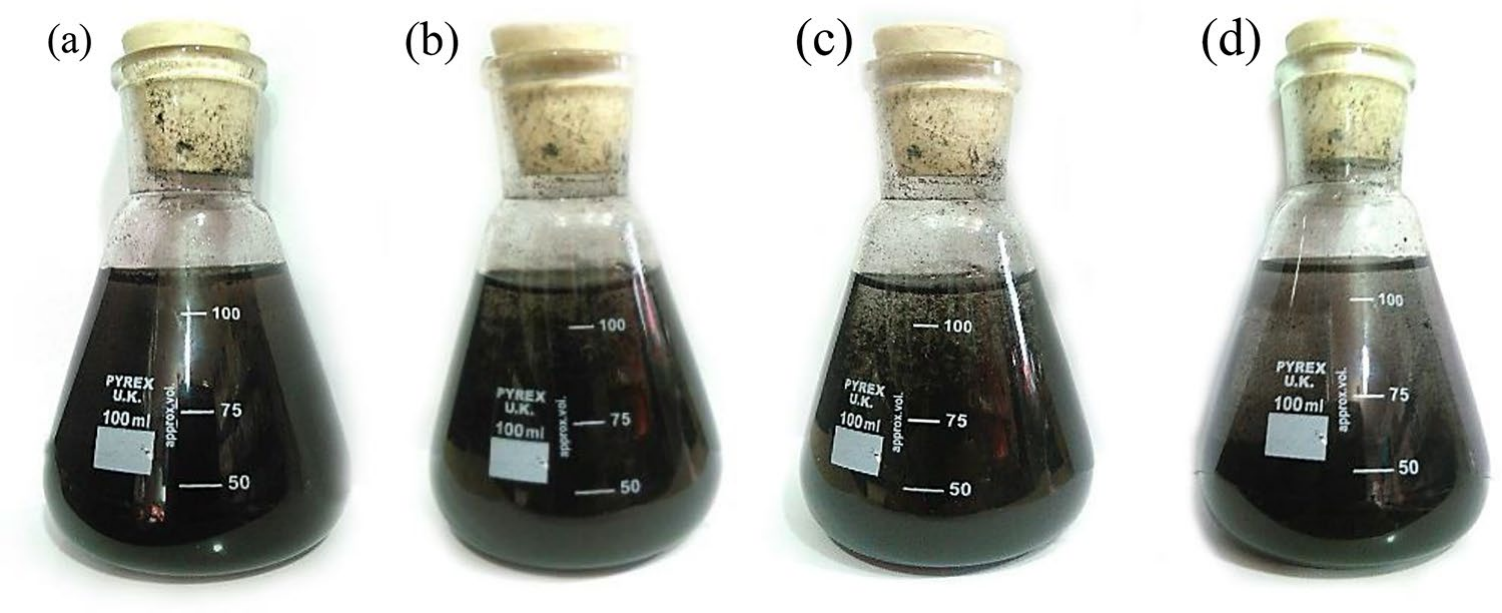
Visualizations of the Fe_3_O_4_/water nanofluid at a concentration of 3 vol% after (**a**) 1 day, (**b**) 2 days, (**c**) 3 days, (**d**) 4 days.

When a magnetic field is not present, ferrofluids exhibit specific interactions with nanofluids, similar to those caused by Brownian motion^53,54^. However, when there is no movement, sedimentation begins to take place. As shown in Fig. 1, the sedimentation process of ferrofluid nanoparticles in the motionless liquid alters over time, culminating in nearly full settling within 24 h. Visual observations indicated that the Fe_3_O_4_/water nanofluid remained remarkably stable for 4 days. As a result, it is reasonable to assume that the nanofluid maintained its stability throughout the experiments.

**Figure 2:**
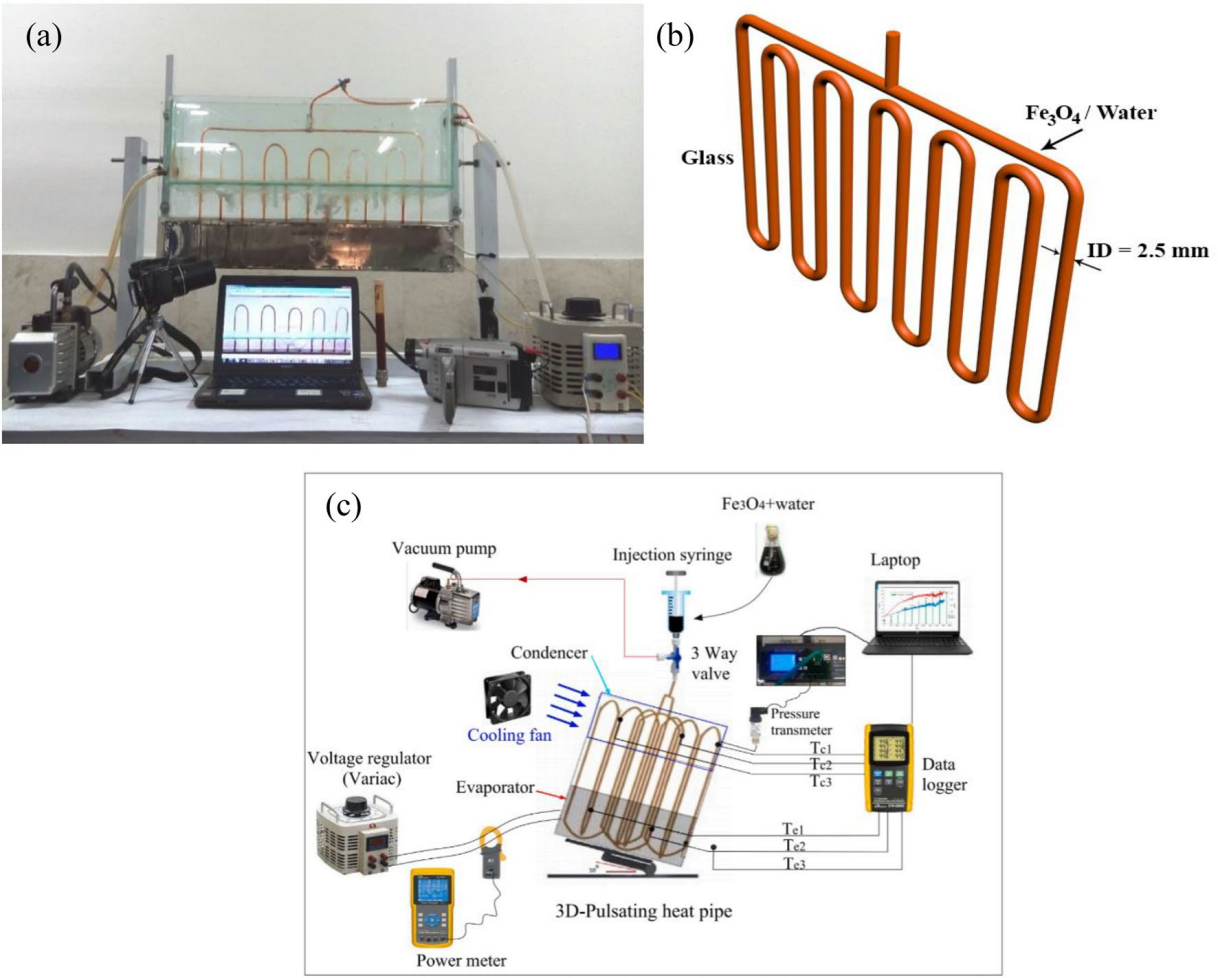
(**a**) Experimental setup, (**b**) schematic of the closed-loop PHP configuration, (**c**) schematic of experimental setup^55,56^.

Figure 2 illustrates images of the experimental arrangement, a schematic representation outlining the configuration of the closed-loop PHP, and a schematic of the experimental setup. The primary goal of this investigation was to assess how varying heat loads affect the evaporator section within the closed-loop PHP. To achieve this objective, a controlled range of heat inputs (ranging from 10 to 80 W) was simulated on the vertical PHP’s evaporator using an electric heater connected to a variac. Standard current and voltage meters were incorporated in this setup. The uncertainties associated with the current and voltage measurements were identified to be ± 0.015 A and ± 0.4 V, respectively. To monitor temperatures at both the condenser and evaporator, a set of K-type thermocouples was employed in conjunction with a data acquisition system. The precision of the temperature measurements was evaluated at ± 1 K. The remaining geometric specifications for the PHP can be found in Table 2.

**Table 2:** Heat pipe configuration.

PHP container	Copper and glass
PHP length	380 mm
Condenser length	100 mm
Adiabatic length	100 mm
Evaporator length	100 mm
Outer diameter	3 mm
Wall thickness	1.25 mm
Inner diameter	1.75 mm
Liquid filled ratio	50%
Total length of PHP	4.4 m

## Methodology and reproducibility

As previously indicated, the hybrid composition employed in this study encompassed Fe_3_O_4_ nanoparticles at a volume concentration of 3%. These nanoparticles were thoroughly mixed with the base fluid through conventional stirring techniques. A uniform mixture was ensured by subjecting the blend to ultrasonic oscillation for a duration of 5 h.

A meticulous evacuation procedure was implemented before introducing the fluid into the PHP. A vacuum pump, connected via a 3-way valve, was used to achieve a suction pressure of 0.1 Pa for a duration of 15 min.

The experimental trials encompassed a range of heat conditions, with heat loads spanning from 10 to 80 W applied to the PHP’s evaporator. Steady-state conditions were determined by considering a temperature fluctuation of less than ± 0.1 °C over a 10-min interval, in accordance with methodologies previously established^53,54^. This approach mirrors the methodology outlined by Li and Yan^57^. Once steady-state conditions were confirmed, the power was systematically incremented to the next level, thereby evaluating the performance of the PHP. This iterative process was replicated for each heat setting, covering the entire spectrum from 10 to 80 W.

After each series of runs, a 2-week idle period was observed before conducting subsequent tests. Impressively, these subsequent tests displayed no discernible deviations in results. The noteworthy depicted consistency can be attributed to the erratic movement of nanoparticles influenced by buoyancy within the base liquid. This likely resulted in the creation of a uniform nanofluid following the extended idle period. This observation aligns with the findings reported by Lu et al.^58^, affirming that tests conducted on uniformly dispersed nanofluids can yield remarkably reproducible outcomes.

## Visualization results

Flow visualization in this study entailed using Pyrex glass (7740) material. Capturing the dynamic phase change phenomena of heating and bubble generation required a high-speed video camera (Panasonic VX87) equipped with a frame rate of 100 frames per second and a sensitivity of 1504 × 1128 pixels. Operating in real-time, the camera facilitated the instant transmission and storage of the recorded images onto a laptop. To optimize visibility, an intense light source was employed to illuminate the flow dynamics within the PHP, thereby enhancing the clarity of the captured images. Each successive image was taken with a time step of 0.007 s, corresponding to the established recording rate of 100 frames per second. The consecutive images were subsequently subjected to processing using commercial software, specifically Photoshop CC. The goal was to measure the distance traversed by the fluid over defined time intervals. This quantification enabled the calculation of velocity by dividing the measured distance by the corresponding time elapsed.

Operating at a heating power of 30 W, the internal flow behavior of the system was meticulously observed. In the initial phases, the majority of liquid slugs settled at the lower sections of the vertically arranged channels, driven by the force of gravity. Bubbles emerged primarily within the side channels. As the temperature within the evaporation section escalated, the bubble growth rate exhibited a proportional increase. This phenomenon led to the acceleration of subsequent bubbles, resulting in the amalgamation of multiple smaller spherical bubbles into more substantial vapor columns.

The extreme boiling characteristics of Fe_3_O_4_/water introduced pressure imbalances, yielding intermittent oscillations characterized by upward and downward motion. An observable number of bubbles surfaced within the middle channels, eventually transitioning the localized oscillation within these channels into a collective oscillation spanning all interconnecting channels.

As the heating input persisted within the evaporator segment, the intermediate bubbles demonstrated accelerated growth within the liquid plugs, outpacing the development of elongated columnar bubbles. This continuous heating instigated the generation of substantial vapor content within the intermediate bubbles, thereby augmenting vapor pressure. Given the overriding influence of surface tension within the PHP, a cyclic sequence of liquid slugs and vapor plugs manifested within the system. Rapid increments in vapor pressure prompted a swift succession of movements for both vapor bubbles and liquid plugs through the capillary^59–62^.

**Figure 3:**
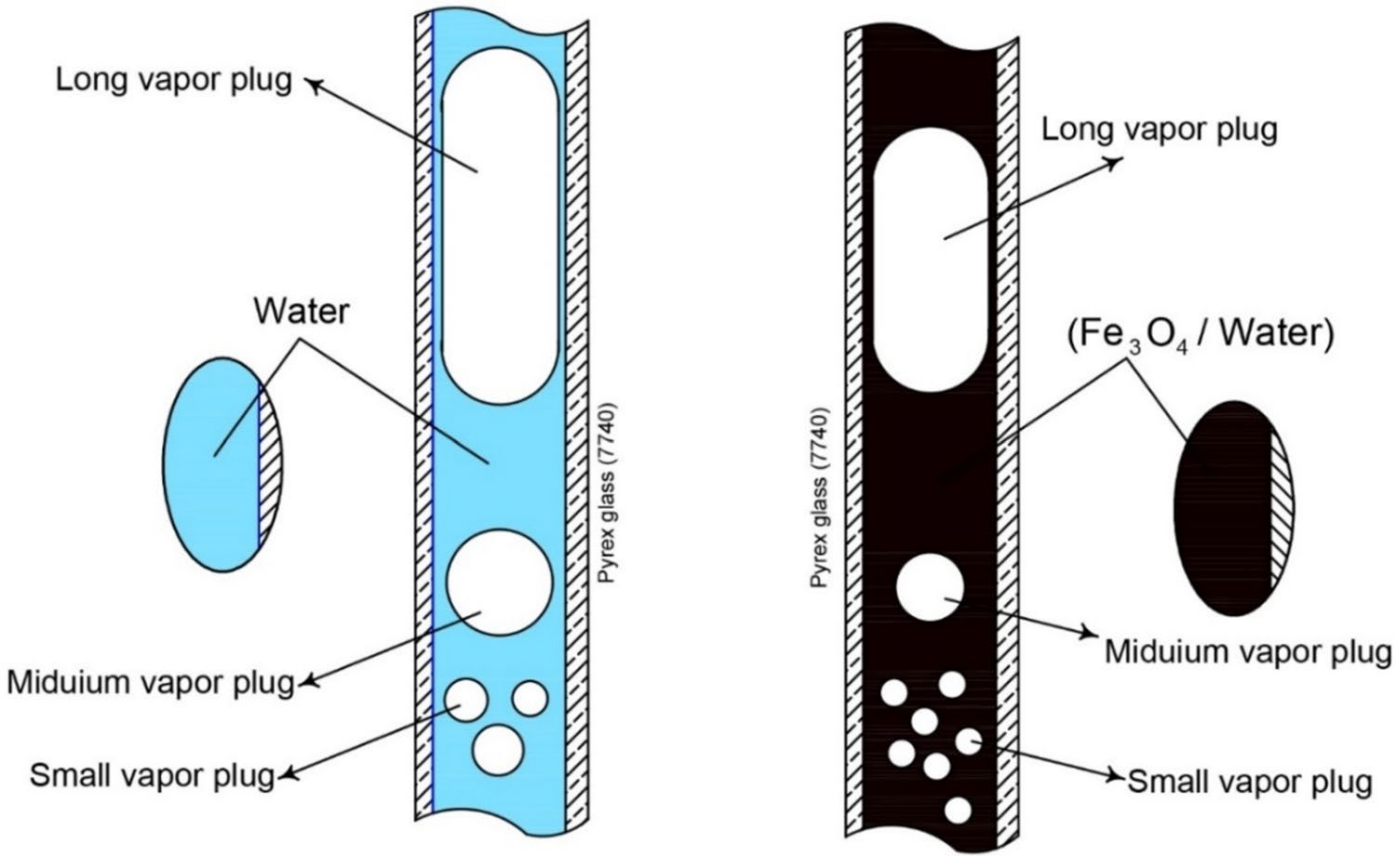
Schematic of three types of bubbles for pure water and Fe_3_O_4_/water.

Crucially, the input heat, functioning as the driving force, elevated the vapor plug pressure within the evaporator, while the heat output simultaneously reduced the vapor plug pressure within the condenser^63^. During the flow visualization phase, it was observed that lower heat inputs at the evaporator corresponded to extended intervals before the onset of nucleate boiling, consequently delaying the abrupt motion of vapor bubbles and liquid plugs within the PHP. Figure 3 effectively illustrates three distinct bubble categories based on size: small bubbles, vapor plugs, and elongated vapor plugs. These phenomena were observable in both pure water and Fe_3_O_4_/water systems. Small bubbles manifested due to persistent nucleate pool boiling in scenarios where coalescence within the evaporator section was absent. Vapor plugs, approximately equal in size to the tube diameter, were the predominant outcome. Additionally, the presence of extended vapor plugs, particularly noticeable in pure water, exhibited susceptibility to collapse. These characteristics were also prominently observed throughout the visualization experiments.

### Flow patterns

**Figure 4:**
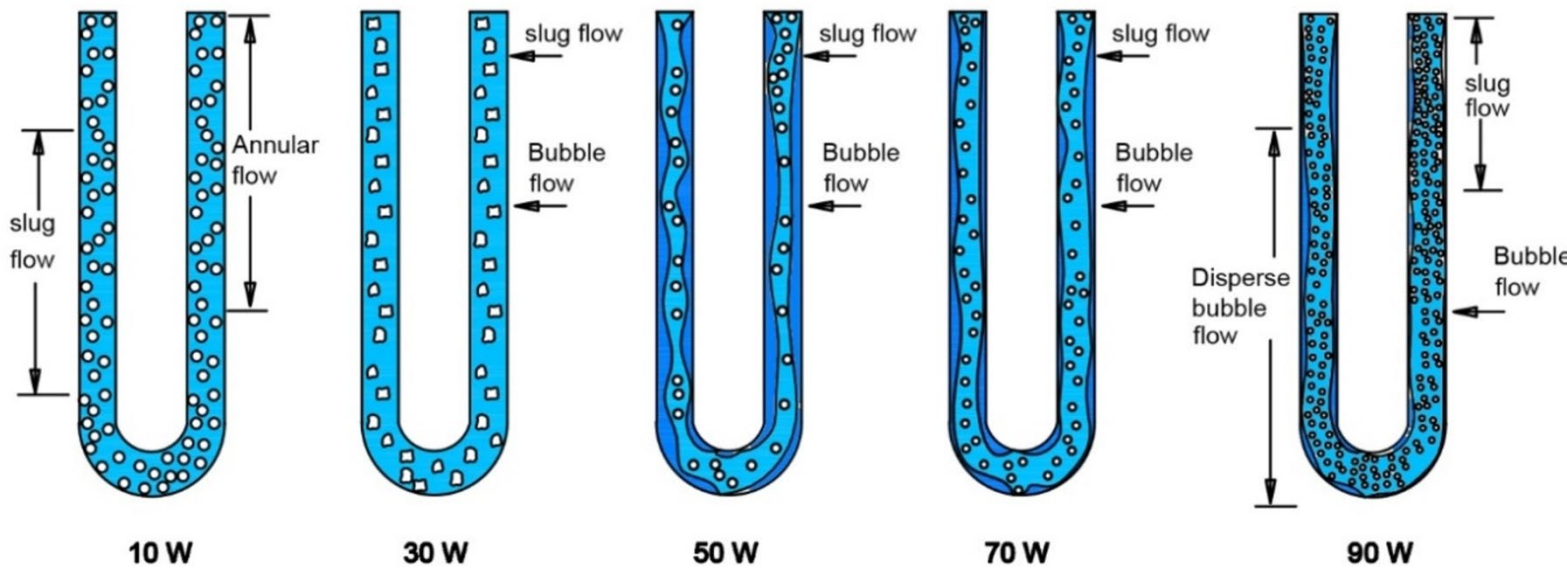
Flow regimes within the evaporator section of the PHP under varying heat loads for the water system.

The arrangement of slugs and plugs was also subject to observation. As presented in Fig. 4, the vertical PHP displayed the simultaneous presence of distinct lengths of liquid slugs and vapor plugs upon the application of heat. This phenomenon arises from the surface tension force being dominant over the gravitational force^64^. Within the evaporator, bubbles materialized due to the rapid evaporation occurring at nucleation sites. These bubbles, once generated, were swiftly conveyed by the robust slug flow’s high velocity coursing through the evaporator. This efficient transport mechanism facilitated substantial heat transfer from the evaporator to the condenser region. This boiling process within the PHP’s evaporator could be classified as forced convective boiling. As the liquid infiltrated the heated domain, bubbles perpetually displaced the superheated liquid film away from the evaporator surface. Figure 4 illustrates the flow dynamics in the evaporator segment of the PHP under a relatively modest heat load of 10 W.

As the input heat amplified in the evaporator, particularly around 30 W, a discernible bubble formation took place within a continuous liquid phase. These bubbles, characterized by a mean size smaller than the tube’s diameter and relatively fewer in number, emerged. At the 50 W heat input level, smaller bubbles emerged in greater quantities. This augmentation in bubble presence induced pronounced agitation and mixing adjacent to the wall, consequently enhancing the PHP’s heat transfer rate as the heat load escalated. Furthermore, upon reaching an input heat of 70 W, bubble quality transitioned to an intermediate phase, prompting the coalescence of smaller bubbles. This coalescence induced an unstable and oscillatory vapor shear onto the liquid–vapor interface, a phenomenon commonly identified as churn flow. With an input heat of 90 W, the flow regime adopted the characteristics of annular flow. This flow pattern entailed the expulsion of liquid from the tube’s center, resulting in a thin film adhering to the tube wall and forming an annular ring of liquid. The annular flow pattern is recognized for exhibiting higher heat transfer coefficients compared to other flow patterns in two-phase flow analyses. In concordance with our study, the annular flow pattern manifested at the elevated input heat of 90 W, directly contributing to an elevated heat transfer coefficient within the evaporator section.

**Figure 5:**
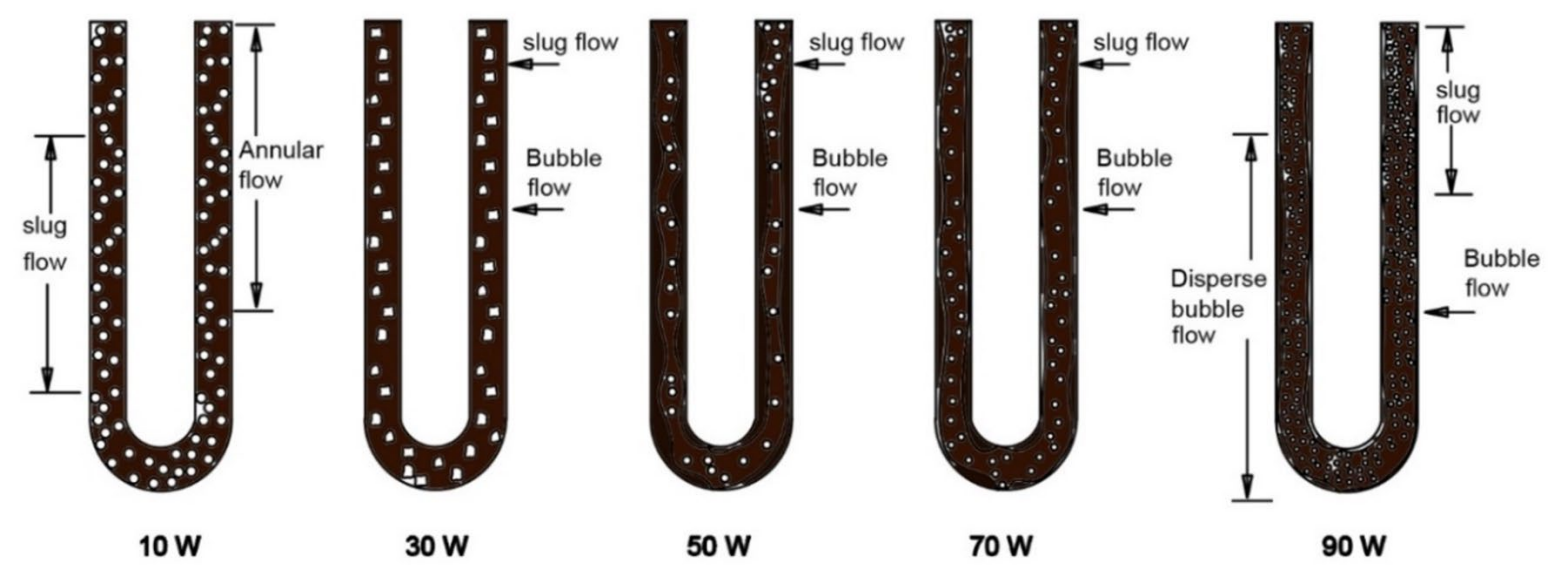
Flow regimes at the evaporator section of PHP under varying heat loads for Fe_3_O_4_/water.

Figure 5 showcases the internal flow patterns observed in the vertical position of the CLPHP/CV employing Fe_3_O_4_/water, providing insights into distinct heat load conditions.

At the modest heat load of 30 W, the observed patterns encompassed annular flow, albeit with only a few nucleation sites. The lower segment of the evaporator displayed slug flow, characterized by a higher nucleation site count. Meanwhile, the middle and upper regions of the evaporator were dominated by slug and annular flow phenomena. Notably, the vapor slug within this context featured a length of approximately 0.03 m, accompanied by a velocity of around 0.1 m/s.

With a heat load of 70 W, slug flow with limited nucleation sites emerged, alongside bubble flow prevalent in the lower evaporator portion marked by numerous nucleation sites. In the middle and upper portions of the evaporator, a coexistence of bubble and slug flow was observable. The vapor slug, during this phase, exhibited dimensions of approximately 0.04 m in length and maintained a velocity around 0.1 m/s.

Upon reaching a substantial heat load of 90 W, the lower evaporator section revealed slug and bubble flow characterized by scarce nucleation sites. Furthermore, dispersed bubble flow, with a more abundant nucleation site distribution, manifested within this region. These vapor-dispersed bubbles propagated towards the middle and upper portions of the evaporator, eventually advancing into the condenser section. The vapor slug, during this scenario, exhibited dimensions of approximately 0.015 m in length, coupled with a velocity of around 0.15 m/s.

### Mapping of flow patterns

**Figure 6:**
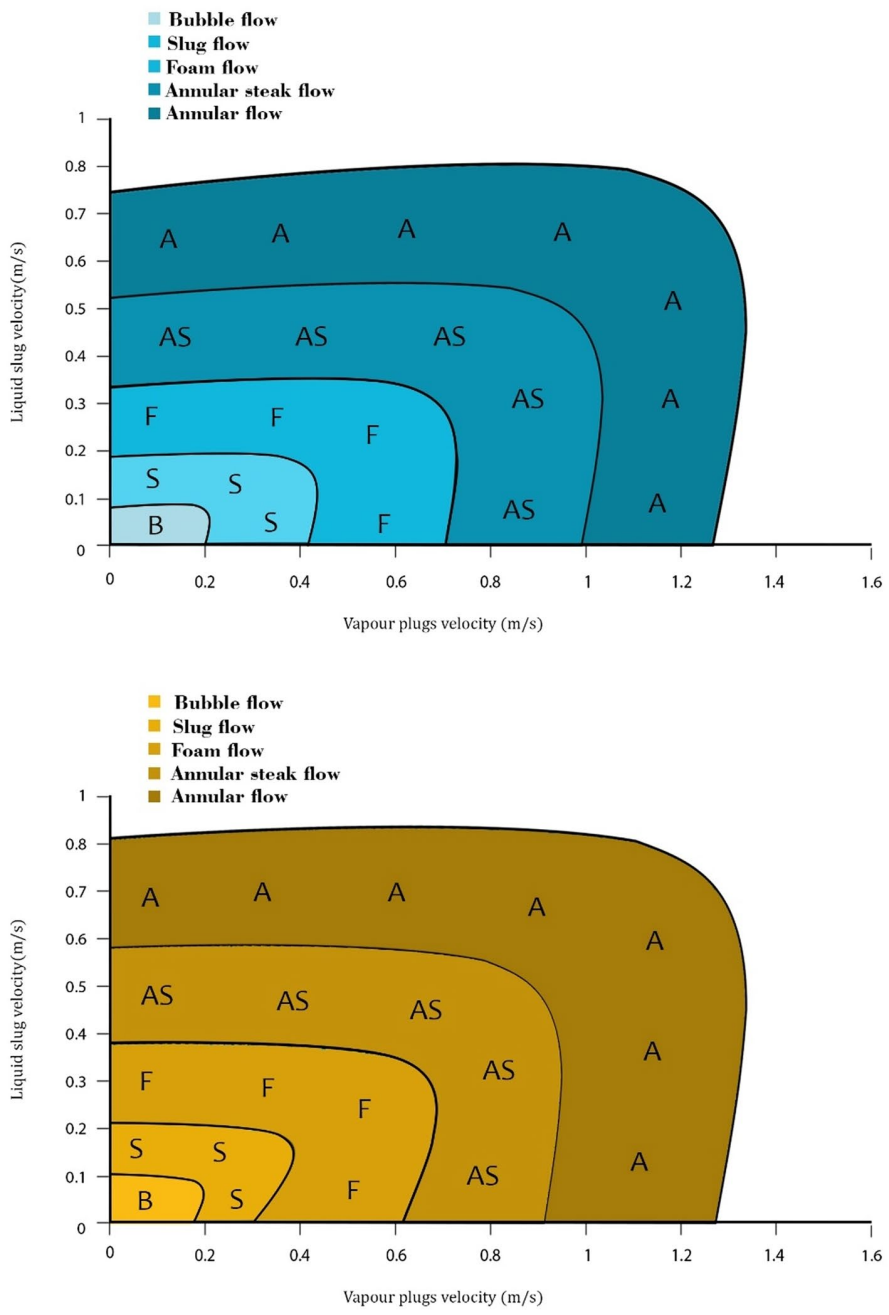
Liquid and vapor plug velocity in the condenser.

The scope of this study extended to a series of experiments involving the PHP in vertical bottom heat mode, with deliberate variations in both the fill ratio (FR) and heat input (q). The comprehensive results were effectively illustrated and interpreted in Fig. 6. Within this graphical representation, the diverse flow patterns were designated by distinct letters: Bubble flow (B), Slug flow (S), Foam flow (F), Annular steak flow (AS), and Annular flow.

The visualization experiments executed within this study showcased that the flow dynamics within the PHP transcended a simplistic single bubble–liquid slug flow model. Instead, the intricacies of flow patterns under varying working conditions unveiled a range of possibilities, encompassing bubble–liquid slug, semi-annular, and annular flow configurations.

However, a comprehensive analysis facilitated the establishment of a direct correlation between the thermal performance of the PHP and the flow patterns occurring within the tube at varying filling ratios. Regions characterized by low filling ratios exhibited a tendency towards bubble flows, while slug flow patterns dominated in instances of higher heat inputs. Remarkably, at an 80% filling ratio, the observation of bubble or slug flows became notably challenging.

The flow patterns associated with a 50% filling ratio showcased an intriguing self-adjusting nature when subjected to escalating heat input. This phenomenon led to a progressive transition from slug flow to semi-annular and ultimately to annular flow. This dynamic shift was notably discerned within tests conducted at a 50% filling ratio and found consistent validation in alignment with prior research findings^61^.

The utilization of a high-speed flow visualization system granted intricate insights into the underlying flow patterns. These patterns were classified into five distinct categories, contingent on the sizes of the bubbles: bubble flow, slug flow, foam flow, annular steak flow, and annular flow. The outcomes of the visualization, portrayed in Fig. 6, could be methodically classified as follows:

Bubbly Flow (B): Vapor bubbles exhibited a remarkable uniformity in size. The formation of these smaller bubbles emanated from continuous nucleate pool boiling, accompanied by detachment from the tube’s inner wall.

Slug Flow (S): The vapor maneuvered through the conduit in the form of substantial bullet-shaped bubbles, akin to vapor plugs. Notably, the extent of the vapor plug varied, encompassing both extended and abbreviated forms. Another variant entailed the emergence of small bubbles, devoid of subsequent growth.

Foam Flow (F): A distinctive flow pattern characterized by significant instability, characterized by oscillatory dynamics. The fluid in proximity to the tube wall underwent perpetual pulsations, alternating between upward and downward movements.

Annular Steak Flow (AS): In the context of annular flow, the escalation in liquid flow rates precipitated a corresponding augmentation in droplet concentration within the vapor core. This gradual accumulation of droplets within the core ultimately yielded prominent accumulations or streaks of liquid materializing within the vapor core.

Annular flow (A) was characterized by the liquid’s bifurcation into a partial annular film tracing the tube walls and small droplets dispersed within the central vapor stream. The flow configuration within a PHP exhibited malleability in response to escalating heat input, transmuting from slug-plug dynamics to an annular flow configuration. The thermal efficacy of the PHP was notably elevated during annular flow^52^.

Nonetheless, within the scope of this experiment, the probability of dry-out occurrences and the escalation of the evaporator’s temperature were notably higher at elevated heat inputs. The heat transfer mechanisms within PHPs were intertwined, incorporating both sensible and latent heat transfer modes, with their relative prominence significantly influencing the holistic thermal performance of the PHP. The slug flow regime primarily manifested sensible heat transfer, whereas latent heat transfer gained supremacy within the annular flow domain^64–66^.

The revelations from visualization underscored the counterclockwise trajectory of the liquid slugs, maintaining a consistent velocity direction. However, modest variations in these values were witnessed in short intervals, albeit with minimal fluctuations in amplitude.

## Conclusions

The primary aim of this study was to assess the impact of various heat loads on the evaporator section of a closed-loop PHP. We simulated a controlled range of heat inputs (10–80 W) on the vertical PHP’s evaporator using an electric heater connected to a variac. This study thoroughly investigated the internal flow dynamics of a closed-loop oscillating heat pipe using a 3% Fe_3_O_4_/water mixture. The predominant flow pattern transitioned from a combination of bubble and slug flow to an annular flow configuration. We identified five distinct flow patterns: Bubbly flow (B), Slug flow (S), Foam flow (F), Annular steak flow (AS), and Annular flow (A). The behavior of the Fe_3_O_4_/water mixture varied with fill ratios from 0 to 80%. Lower fill ratios led to fewer bubbles and shorter slug lengths, while higher temperatures in the lower section formed larger vapor slugs. Optimal generation of liquid slugs and vapor bubbles required selecting internal tube diameters (0.1–5 mm) and maintaining slug velocity below 0.1 m/s to prevent small bubbles from detaching from the tube wall. Several significant conclusions were drawn from the experimental results and performance discussions:

1. The vapor slug length decreased rapidly with increasing slug velocity, facilitating the determination of the main regime for each flow pattern using the proposed map.
2. Fe_3_O_4_/water mixture exhibited reduced dimensions for liquid and vapor slugs compared to pure water, attributed to higher non-dimensional parameters specific to acetone, indicating more favorable fluid flow within the PHP.
3. The internal fluid flow in the PHP was found to be more complex than single bubble–liquid slug flow, with various distinct flow patterns observed, contributing to an enhanced net heat transfer coefficient.
4. PHPs displayed self-adjusting characteristics for flow patterns to meet increasing heat input demands, notably demonstrated in 50% filling ratio tests.
5. The dynamic circulation of Fe_3_O_4_/water in the PHP facilitated efficient heat transfer from the evaporator to the condenser, showcasing diverse phase change phenomena in the heating section.
6. Fe_3_O_4_/water velocity correlated directly with heat input magnitude, influencing prevailing flow patterns within the PHP significantly.

## Data Availability

All data generated or analyzed during this study are included in this published article.
