# Author Correction: Insights into two-phase flow dynamics in closed-loop pulsating heat pipes utilizing Fe_3_O_4_/water: experimental visualization study

**DOI:** 10.1038/s41598-024-72301-6

**Published:** 2024-09-10

**Authors:** Hamid Reza Goshayeshi, Seyed Borhan Mousavi, Saeed Zeinali Heris, Issa Chaer

**Affiliations:** 1 School of Safety Science and Engineering, Xi’an University of Science and Technology, 58, Yanta Mid. Rd., Xi’an, 710054 Shaanxi China; 2 Department of Mechanical Engineering, Mashhad Branch, Islamic Azad University, Mashhad, Iran; 3 J. Mike Walker ‘66 Mechanical Engineering Department, Texas A&M University, College Station, TX 77843 USA; 4 Faculty of Chemical and Petroleum Engineering, University of Tabriz, Tabriz, Iran; 5 School of Built Environment and Architecture, London South Bank University, London, SE1 0AA UK

Correction to: *Scientific Reports* 10.1038/s41598-024-67637-y, published online 17 July 2024

The original version of this Article omitted an affiliation for Saeed Zeinali Heris. The correct affiliations are listed below.

School of Safety Science and Engineering, Xi’an University of Science and Technology, 58, Yanta Mid. Rd., Xi’an 710054, Shaanxi, China.

Faculty of Chemical and Petroleum Engineering, University of Tabriz, Tabriz, Iran.

The original Article has been corrected.

